# Multimodal magnetic resonance imaging on brain structure and function changes in vascular cognitive impairment without dementia

**DOI:** 10.3389/fnagi.2023.1278390

**Published:** 2023-11-16

**Authors:** Qinhong Zhang, Xiao Liu, Shenglan Gao, Shiyan Yan, Ang Li, Zeyi Wei, Shengwang Han, Yu Hou, Xiaoling Li, Danna Cao, Jinhuan Yue

**Affiliations:** ^1^Shenzhen Frontiers in Chinese Medicine Research Co., Ltd., Shenzhen, China; ^2^Department of Acupuncture and Moxibustion, Heilongjiang University of Chinese Medicine, Harbin, China; ^3^Department of Pediatrics, First Affiliated Hospital of Heilongjiang University of Chinese Medicine, Harbin, China; ^4^Graduate School of Heilongjiang University of Chinese Medicine, Harbin, China; ^5^School of Acupuncture-Moxibustion and Tuina, Beijing University of Chinese Medicine, Beijing, China; ^6^Servier (Beijing) Pharmaceutical Research and Development Co., Ltd., Beijing, China; ^7^Third Ward of Rehabilitation Department, Second Affiliated Hospital of Heilongjiang University of Chinese Medicine, Harbin, China; ^8^Department of Gynecology, Harbin Traditional Chinese Medicine Hospital, Harbin, China; ^9^Division of CT and MRI, First Affiliated Hospital of Heilongjiang University of Chinese Medicine, Harbin, China; ^10^Department of Acupuncture and Moxibustion, Vitality University, Hayward, CA, United States

**Keywords:** vascular cognitive impairment not dementia, multimodal, magnetic resonance imaging, brain structure, brain function

## Abstract

Vascular cognitive impairment not dementia (VCIND) is one of the three subtypes of vascular cognitive impairment (VCI), with cognitive dysfunction and symptoms ranging between normal cognitive function and vascular dementia. The specific mechanisms underlying VCIND are still not fully understood, and there is a lack of specific diagnostic markers in clinical practice. With the rapid development of magnetic resonance imaging (MRI) technology, structural MRI (sMRI) and functional MRI (fMRI) have become effective methods for exploring the neurobiological mechanisms of VCIND and have made continuous progress. This article provides a comprehensive overview of the research progress in VCIND using multimodal MRI, including sMRI, diffusion tensor imaging, resting-state fMRI, and magnetic resonance spectroscopy. By integrating findings from these multiple modalities, this study presents a novel perspective on the neuropathological mechanisms underlying VCIND. It not only highlights the importance of multimodal MRI in unraveling the complex nature of VCIND but also lays the foundation for future research examining the relationship between brain structure, function, and cognitive impairment in VCIND. These new perspectives and strategies ultimately hold the potential to contribute to the development of more effective diagnostic tools and therapeutic interventions for VCIND.

## Introduction

Vascular cognitive impairment (VCI) is a recognized disease primarily caused by cerebrovascular diseases (Corriveau et al., 2016; Rundek et al., 2022). VCI-non-dementia (VCIND) is the mildest stage of VCI and the most common type among elderly individuals, with a diagnosis rate of 2.4% in the population aged 65 years and above. It is also associated with an increased risk of mortality (Rockwood et al., 2000). Patients with VCIND may experience impairments in multiple or single cognitive domains, such as visual–spatial and executive abilities, memory, attention, language, abstract thinking, calculation, and orientation (Sun, 2018; van der Flier et al., 2018). The progression of VCIND can be insidious and slow, often accompanied by cognitive impairments in multiple brain regions (Li et al., 2023). Existing evidence suggests that the progression of VCIND can be delayed or even reversed through early detection and intervention (Gorelick et al., 2011).

With the continuous development of multimodal magnetic resonance imaging (MRI) techniques, including structural MRI (sMRI), diffusion tensor imaging (DTI), resting-state functional MRI (rs-fMRI), and hydrogen proton magnetic resonance spectroscopy (^1^H-MRS), it is possible to obtain brain structural, functional, and metabolic changes to evaluate or predict abnormal changes in the brain structure and function in VCIND, thereby providing assistance for the study of central mechanisms in VCIND (Gorelick et al., 2011).

### VCIND study of brain structure

#### SMRI

**Figure 1:**
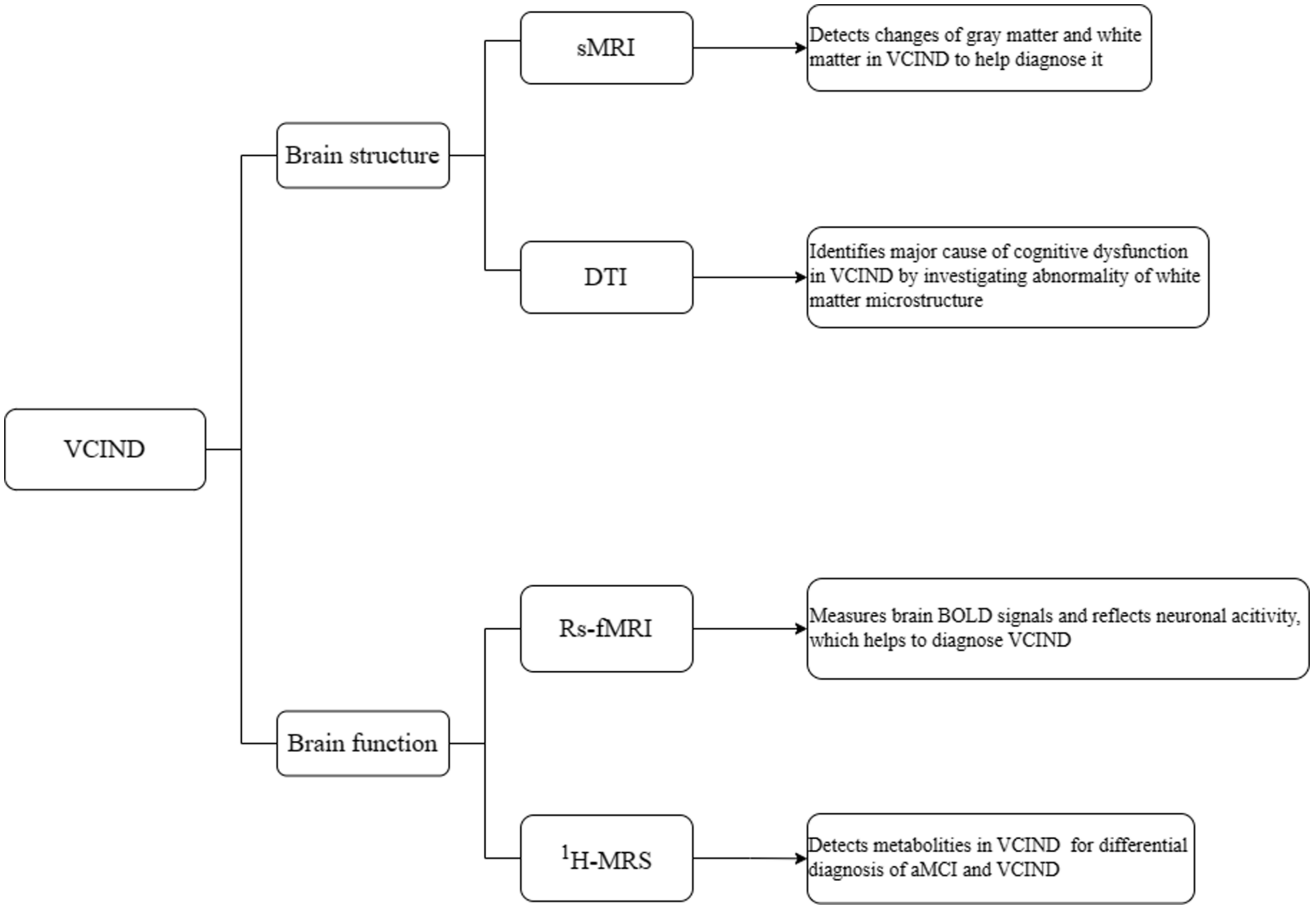
Multimodal MRI of brain structure and function in VCIND.

SMRI is a non-invasive clinical imaging technique commonly used to examine the anatomical structure of the brain, as well as to identify diseases through examining morphological changes (Figure 1) (Frisoni et al., 2010; Vemuri and Jack Jr, 2010). Tan et al. (2023) conducted a study using gray matter density analysis to investigate differences in gray matter volume between healthy controls and patients with subcortical ischemic vascular dementia (SIVD), including those with normal cognition, VCIND, and vascular dementia (VaD). The results showed that, in the SIVD-VCIND group, there was a positive correlation between gray matter total volume, right temporal superior gyrus, middle frontal gyrus, anterior cingulate gyrus, bilateral orbital medial gyrus, and Boston Naming Test scores, as well as a positive correlation between gray matter total volume, right superior frontal gyrus, right middle frontal gyrus, and overall cognitive function. These findings reveal the relationship between structural imaging changes caused by SIVD-VCIND and cognitive impairment, providing valuable evidence for understanding the pathogenesis of subcortical VCI and aiding in the early diagnosis of SIVD-VCIND and early warning of SIVD-VaD. Zhao et al. (2022) investigated the value of brain sMRI combined with APOE-ε4 genotype for the early diagnosis and progression of VCIND in elderly patients. It was found that the VCIND group had significantly higher white matter (WM) volume (WMV), WM hyperintensity (WMH), and Fazekas scores compared to the normal control group. Logistic regression analysis results showed that patients with higher WMV, WMH, and Fazekas scores had a higher probability of developing VCIND. The follow-up results of VCIND showed that, compared to the non-dementia group, the dementia group had significantly higher WMV, WMH, and Fazekas scores. These results suggest that widespread injury caused by white matter lesions can lead to VCIND. Zhang et al. (2022) investigated the application of MRI combined with neuropsychological assessment based on artificial intelligence technology in cognitive function impairment in patients with neurovascular diseases. The patients were divided into control, VCIND, VaD, and Alzheimer’s disease (AD) groups. All patients underwent MRI, neuropsychological evaluations, and examinations, and an improved fuzzy c-means (FCM) clustering algorithm was proposed for MRI processing. The study found that the segmentation accuracy (SA) and similarity index of the improved FCM algorithm were higher than those of the standard FCM algorithm, bias-corrected FCM algorithm, and rough FCM algorithm. In terms of activities of daily living (ADL), the VCIND group and VD group were higher than the control group, indicating a negative correlation between hippocampal volume and ADL. In conclusion, the improved FCM algorithm has higher segmentation effectiveness and SA in MRI of neurovascular diseases. Additionally, the distribution, quantity, WM lesions, and hippocampal volume of lacunar infarction are associated with cognitive function impairment in patients with cerebrovascular diseases.

#### DTI

DTI is an advanced MRI technique that measures the directionality and magnitude of water molecules diffusion in neural tissue to reflect the microstructural integrity of WM tracts (Douglas et al., 2015; Antkowiak et al., 2021). The commonly reported DTI indices include fractional anisotropy (FA) and mean diffusivity (MD), which represent the degree of directionality and average diffusivity of water, respectively (Mayo et al., 2019; Dorsman et al., 2020; Kimura et al., 2020). Qin et al. (2021) applied DTI technique to investigate the changes in the microstructure of default mode network (DMN) WM in patients with VCIND and its role in cognitive dysfunction. Compared to healthy elderly individuals, patients diagnosed with subcortical VCIND showed abnormal WM integrity in several key hubs of the DMN. The severity of DMN WM microstructural damage was significantly associated with cognitive impairment. The integrity of DMN in patients with subcortical VCIND was significantly compromised, and the disruption of DMN connectivity could explain the impairments in attention, language, and executive functions, suggesting that the WM integrity of DMN may serve as a neuroimaging indicator for VCIND. In their study, Zhou et al. (2011) focused on using DTI histogram analysis to detect WM damage in patients with VCIND, and they aimed to investigate the correlation between DTI histogram measurements and cognitive impairment in these patients. To conduct their research, the researchers recruited both VCIND patients and cognitively normal individuals as a control group. They analyzed and compared the MD and FA histograms of WM and normal appearing WM (NAWM) for each subject. The results of their analysis revealed that the VCIND group exhibited decreased FA values throughout the brain when compared to the control group. This finding suggests that there is a widespread disruption of WM integrity in VCIND patients. Additionally, significant differences were observed in the FA and MD histogram patterns between the VCIND group and the control group for both WM and NAWM. To further investigate the relationship between DTI measurements and cognitive impairment, the researchers examined the neurocognitive results, measured as z-scores, and found significant correlations with various DTI histogram measurements. Specifically, the average FA peak position, average MD, and average MD peak position of WM were all significantly correlated with the neurocognitive results. Similarly, the average FA peak height, average MD, and average MD peak position of NAWM were also significantly correlated with the neurocognitive outcomes. These findings suggest that the severity of WM and NAWM damage, as indicated by DTI histogram measurements, may be closely related to the level of cognitive impairment in VCIND patients. Overall, the findings of this study indicate that DTI histogram analysis can provide valuable insights into the extent and patterns of WM damage in VCIND patients. Furthermore, the study highlights the potential role of DTI histogram analysis in enhancing our understanding of the pathophysiology of VCIND and its association with cognitive impairment. Chen et al. (2018) conducted DTI examinations on 24 patients with SIVD, including 13 cases of VCIND and 11 cognitively normal individuals. They found widespread reduction in FA and increase in MD values in the WM areas of the VCIND group, primarily in the corpus callosum, bilateral internal capsule/radiation of the corona radiata/thalamus posterior region/frontal–parietal subcortical pathway, and the right inferior/superior longitudinal fasciculus. The distribution of areas with decreased FA and lower local diffusion homogeneity (LDH) values showed slight differences. There was a positive correlation between the values of WM, FA (*r* = 0.653, *p* = 0.001), and LDH (*r* = 0.617, *p* = 0.001) levels and the cognitive test results in patients with SIVD. On the other hand, there was a negative correlation between the WMMD (*r* = −0.597, *p* = 0.002) value and the cognitive test scores in patients with SIVD. These findings indicate that abnormal WM microstructure is an important factor contributing to cognitive dysfunction in patients with SIVD, and DTI parameters may serve as potential biomarkers for detecting VCIND in SIVD (Figure 1).

### VCIND study of brain function

#### Rs-fMRI

FMRI measures the blood oxygen level-dependent (BOLD) signal in the brain, which is determined by the levels of oxygenated and deoxygenated hemoglobin and reflects neuronal activity (Li et al., 2018; Ryan et al., 2023). Rs-fMRI measures low-frequency BOLD signals. It estimates the brain’s BOLD signal in awake participants when they are not performing any specific tasks (Azeez and Biswal, 2017; Wei et al., 2022). Wang et al. (2019) divided patients with brain injuries into two groups: those with white matter lesions and VCIND (WMLs-VCIND) and those with WMLs-VaD. Rs-fMRI data were collected and it was found that the overall functional connectivity strength was lowest in WMLs-VaD patients and highest in normal control participants. Both the WMLs-VCIND group and the WMLs-VaD group exhibited a decrease in small-world characteristics compared to the normal control group. Furthermore, the small-world properties were significantly correlated with the Montreal Cognitive Assessment scores. This suggests a potential constructive reorganization of brain networks after brain injury and provides new insights into the role of small-world characteristics (a small-world network is a form that lies between random networks and regular networks, with relatively shorter average path lengths and higher clustering efficiency) in cognitive impairment following brain damage. Sun et al. (2011) conducted a study in which they obtained rs-fMRI data from patients with SIVD who met the criteria for VCIND, as well as a control group of SIVD patients without cognitive impairment. They utilized a time-correlation method to investigate the synchronized low-frequency fMRI signal fluctuations to assess connectivity within the brain. Comparing the VCIND patients to the control group, the researchers discovered decreased FCin regions such as the left middle temporal gyrus, left anterior cingulate gyrus/left middle frontal gyrus, right caudate nucleus, right middle frontal gyrus, and left medial orbital gyrus/paracentral lobule. On the other hand, they observed increased connectivity in certain regions like the right inferior temporal gyrus, left middle temporal gyrus, left precentral gyrus, and left superior parietal lobule. These findings revealed alterations in the neural activity patterns during resting-state in VCIND patients. The changes in connectivity observed in VCIND patients are likely associated with subcortical WM lesions, which disrupt both direct and indirect fiber bundle connections in the brain’s WM. These lesions also impact cortical FC and are influenced by decreased perfusion due to small vessel disease. Overall, the simplicity and non-invasiveness of this method suggest its potential as a valuable tool in understanding the underlying mechanisms of VCIND. Shi et al. (2020) divided participants into three groups: the group with lacunar infarction and VaD (LA-VaD), the group with lacunar infarction and VCIND (LA-VCIND), and the normal control group. They used independent component analysis and Granger causality analysis to study changes in resting-state networks (RSN). The functional connectivity strength of the networks varied between the normal control group, the LA-VCIND group, and the LA-VaD group. The effective connectivity between RSNs was compensated by the increase or decrease in effective connectivity changes in these three groups. The composition of resting-state networks continuously changed with the progression of the disease. This indicates that the human brain compensates for specific functional changes at different stages (Figure 1).

#### ^1^H-MRS

MRS can be used in conjunction with conventional MRI to characterize the tissues of living animals (Rhodes, 2017). Unlike MRI, MRS does not produce images but rather generates spectra, where different peaks can be distinguished and attributed to different chemical groups and metabolites, enabling non-invasive quantification of metabolites (van de Weijer and Schrauwen-Hinderling, 2019). Depending on the acquisition method, several low molecular weight metabolites can be detected, such as N-acetyl aspartate (NAA), choline-containing compounds (Cho), creatine and phosphocreatine (Cr), myo-inositol (mI), and glutamate (Glu) (Baruth et al., 2013; Harris et al., 2017). NAA represents the integrity of neurons and axons, and a decrease in its content may indicate neuronal tissue loss or damage. Alterations in Cho levels can indicate cellular proliferation, changes in cell membrane phospholipid turnover, or inflammation (Oz et al., 2014), while Cr concentration is often considered constant, it’s important to note that differences in Cr concentration may also occur. MI has regulating functions in osmotic pressure, cell nutrition, antioxidant effects, and the production of surfactants. Glu is a key metabolite due to its role as the primary excitatory neurotransmitter in the central nervous system. Quantifying Glu levels using ^1^H MRS offers insights into neuronal and synaptic integrity and the metabolic processes associated with glutamatergic neurotransmission.

^1^H-MRS is the most abundant atomic nucleus in human body, so the application of ^1^H-MRS is the most extensive. Chen et al. (2016) conducted hydrogen proton ^1^H-MRS examinations on patients with multiple-domain amnestic mild cognitive impairment (M-aMCI) and VCIND. They measured levels of NAA, Glu, mI, Cho, and creatine (Cr). Compared to the normal control group, the NAA/Cr ratio in all regions of interest was significantly reduced in both the M-aMCI and VCIND groups. The Glu-Cr ratio in the posterior cingulate gyrus was significantly lower in the M-aMCI group compared to VCIND. The mI-Cr ratio in the frontal white matter was significantly higher in the VCIND group compared to M-aMCI. These results suggest that ^1^H-MRS is an effective method for differentiating between M-aMCI and VCIND. Liu et al. (2014) used two-dimensional chemical shift imaging proton MRS to evaluate and characterize the metabolic markers of aMCI and VCIND patients compared to normal control subjects. The NAA/Cho ratio in the bilateral white matter of frontal lobe (FLWM), left occipital lobe white matter, and right dorsal thalamus (DT) was significantly lower in VCIND patients compared to normal control or aMCI patients. Furthermore, compared to the control group, VCIND patients exhibited decreased NAA/Cr values in the bilateral DT and FLWM. In addition, aMCI patients showed increased mI in the right posterior cingulate gyrus, and VCIND patients showed increased Cho in the left FLWM. These findings may contribute to the clinical differentiation of these two diseases (Figure 1).

## Limitations

There are two limitations in the current research. Firstly, the heterogeneity of results may be caused by differences in image quality, magnetic field strength, characteristics of participants (age, gender, education, etc.), and analysis methods in different studies. Secondly, some studies have small sample sizes. In the future, larger samples and more comprehensive and homogeneous evaluation indicators can be used for in-depth analysis of VCIND. It is believed that as research continues to deepen, multimodal MRI will make greater progress in the diagnosis and treatment of VCIND.

## Summary

In summary, various multimodal MRI methods, such as sMRI, DTI, rs-fMRI, and ^1^H-MRS examination methods focus on different aspects in VCIND, but all can reveal the correlation between cognitive impairment and structural and functional changes in the brain. MRI has been widely applied in the exploration of VCIND, and various MRI techniques can detect structural and functional changes in the brain, assisting in early diagnosis and providing imaging evidence for clinical treatment to delay further cognitive deterioration and improve patient survival. SMRI can observe extensive damage to white matter lesions that can lead to VCIND. DTI can indicate whether the white matter fibers of the DMN are intact, which may be a neuroimaging marker for VCIND, while parameters such as FA and MD can serve as potential biomarkers for detecting VCIND. Rs-fMRI suggests that the composition of the brain’s resting-state networks changes continuously with disease progression, indicating that the brain compensates for specific functional changes at different stages. MRS can differentiate between aMCI and VCIND by evaluating metabolic markers in brain tissue.

## Author contributions

QZ: Conceptualization, Data curation, Resources, Validation, Visualization, Writing – original draft, Writing – review & editing. XL: Resources, Validation, Visualization, Writing – review & editing. SG: Conceptualization, Resources, Visualization, Writing – review & editing. SY: Methodology, Resources, Validation, Visualization, Writing – review & editing. AL: Methodology, Software, Validation, Visualization, Writing – review & editing. ZW: Resources, Validation, Visualization, Writing – review & editing. SH: Software, Validation, Visualization, Writing – review & editing. YH: Validation, Visualization, Writing – review & editing. XL: Conceptualization, Data curation, Funding acquisition, Investigation, Project administration, Supervision, Validation, Visualization, Writing – original draft, Writing – review & editing. DC: Conceptualization, Funding acquisition, Investigation, Project administration, Supervision, Validation, Visualization, Writing – review & editing. JY: Conceptualization, Data curation, Project administration, Resources, Supervision, Validation, Visualization, Writing – original draft, Writing – review & editing.

## Glossary

VCIND: vascular cognitive impairment not dementia
VCI: vascular cognitive impairment
VaD: vascular dementia
^1^H-MRS: hydrogen proton magnetic resonance spectroscopy
MRI: magnetic resonance imaging
sMRI: structural MRI
fMRI: functional MRI
DTI: diffusion tensor imaging
rs-fMRI: resting-state fMRI
MRS: magnetic resonance spectroscopy
SIVD: subcortical ischemic vascular dementia
WMV: white matter volume
WMH: white matter hyperintensity
FA: fractional anisotropy
MD: mean diffusivity
DMN: default mode network
AD: Alzheimer’s disease
FCM: fuzzy c-means
SA: segmentation accuracy
ADL: activities of daily living
NAWM: normal appearing WM
LDH: local diffusion homogeneity
BOLD: blood oxygen level-dependent
WMLs: white matter lesions
LA: lacunar infarction;
RSN: resting-state networks
NAA: N-acetyl aspartate, Cho, choline-containing compounds
Cr: creatine and phosphocreatine
mI: myo-inositol; Glu, glutamate
aMCI: amnestic mild cognitive impairment
M-aMCI: multiple-domain aMCI
FLWM: white matter of frontal lobe
DT: dorsal thalamus
